# Barriers to mental health services among at-risk and symptomatic adolescents in the community

**DOI:** 10.1186/s13034-025-00978-2

**Published:** 2025-11-05

**Authors:** Kate R. Kuhlman, Paul Delacruz, Silvestre Lopez, Mai-Lan M. Tran, Emma L. Rodgers

**Affiliations:** 1 Department of Psychology, School of Social Ecology, University of California Irvine, 4546 Social & Behavioral Sciences Gateway, Irvine, CA 92697 USA; 2 Institute for Interdisciplinary Salivary Bioscience, University of California Irvine, Irvine, CA USA; 3 Department of Population Health & Disease Prevention, School of Population and Public Health, University of California Irvine, Irvine, CA USA; 4 Cousins Center for Psychoneuroimmunology, Semel Institute for Neuroscience and Human Behavior, University of California Los Angeles, Los Angeles, CA USA; 5 Project Youth OC, Santa Ana, CA USA

## Abstract

**Background:**

Adolescence is a sensitive period of social, biological, and cognitive development. Prolonged suffering from psychiatric symptoms during this important phase of development has well-established social, health, and occupational costs in adulthood. The purpose of this study was to identify the barriers to engaging in mental health treatment among symptomatic, community-dwelling adolescents.

**Methods:**

In a cross-sectional study, barriers to mental health service use and psychiatric symptoms, including depressive symptoms, anxiety symptoms, and sleep disturbance, were self-reported by 277 adolescents (age 15.14 ± 2.21 years; 52% female; 93.5% Hispanic or Latino).

**Results:**

Clinically elevated psychiatric symptoms were common in this sample; 26% self-reported clinically significant depressive symptoms, 41% self-reported clinically significant anxiety symptoms, and 46% self-reported clinically significant sleep disturbance. Having clinically elevated symptoms of anxiety was associated with more barriers overall, *p* < 0.001. Having more exposure to early life adversity (ELA), being in high school or college relative to middle school, and having clinically elevated symptoms of anxiety were each independently associated with more cost concerns, ELA *p* = 0.02, school *p* = 0.01, anxiety *p* = 0.006. Adolescents in this sample with clinically elevated psychiatric symptoms disproportionately reported barriers to seeking mental health treatment pertaining to not wanting to discuss their mental health concerns with a physician, depression *p* < 0.001 and sleep disturbance *p* = .005, not knowing how to access a mental health provider, anxiety *p* < 0.001, and feeling like their symptoms were not pathological given their current circumstances, for both depressive and anxiety symptoms *p* < 0.001.

**Conclusions:**

Among at-risk youth—predominantly Hispanic/Latino—both intrinsic (normalization, fear/limited knowledge) and extrinsic (cost, transportation, physician referral) barriers shape perceived access to care, with anxiety and sleep problems most strongly tied to overall barriers. Social ecological approaches to addressing these barriers may reduce the time at-risk adolescents delay seeking support or intervention.

Recent estimates suggest that 22.2% of adolescents, constituting millions of youth, suffer with a psychiatric disorder, most commonly an anxiety, mood, or behavior disorder [1]. Among these adolescents, only a minority (~ 38%) will receive any treatment for their symptoms [2], and suffering with psychiatric symptoms and disorders in adolescence leads to reverberating costs well into adulthood, including lower academic achievement, higher rates of unemployment, and lower household incomes [3–5]. Identifying barriers to mental health service utilization among adolescents at-risk for or already experiencing symptoms of a psychiatric disorder is essential to reducing the burden of psychiatric illness on young people, their families, and the healthcare system. The barriers facing symptomatic adolescents today are both extrinsic and intrinsic [6]. Extrinsic factors include low availability of mental health professionals locally, high costs, and transportation-related barriers; intrinsic barriers include a lack of awareness that their suffering warrants additional support, and fears of judgment or social repercussions within their family or peer group (see [7, 8] for reviews). Well-established predictors of barriers to mental health service use among adolescents include younger age, being female, having more severe symptoms of depression, anxiety, or substance use, family stigma, and lower parent education (see [8, 9] for reviews).

Some communities, and the adolescents within them, face disproportionate risk for psychiatric disorders and their symptoms as well as additional barriers to seeking or receiving treatment [9–11]. A systematic review of studies investigating barriers among racial and ethnic minority adolescents noted that Hispanic/Latino populations have been the focus of fewer than 19% of studies [9], despite comprising more than 25% of the adolescent population in the U.S. [12]. Nonetheless, barriers to mental health service utilization among Hispanic/Latino youth that cut across social-ecological levels were identified, including stigma from parents, language barriers, distrust of healthcare institutions, acculturative stress, cost, and transportation [9]. In addition to increasing the representation of Hispanic/Latino youth in literature on barriers to mental health service use, there is also a paucity of literature addressing which barriers are specific to teens with psychiatric need. The vast majority of studies on barriers among youth (~ 70%) used samples with elevated psychiatric symptoms or diagnosed psychopathology [9], but provided limited information on whether some barriers are more influential within specific clinical populations. By contrast, the remaining studies have identified barriers more broadly in youth populations (e.g., parent stigma, socioeconomic factors, and cultural attitudes) without regard for whether these barriers are salient to individuals with psychiatric need.

 The present study determined the unique association between clinically elevated symptoms of anxiety, depression, sleep disturbance and self-reported intrinsic and extrinsic barriers to mental health service use in a high-risk sample of predominantly Hispanic/Latino adolescents. In order to address these gaps in knowledge, the present study aimed to identify the perceived barriers to mental health service utilization among a sample of adolescents experiencing psychiatric symptoms. To this end, the role of demographic (gender, grade in school), psychosocial (parent English fluency, early life adversity exposure), and symptom factors (anxiety, depression, or sleep disturbance) were examined as predictors of barriers to service use both independently and simultaneously. This study extends the existing literature by examining barriers to service use among a predominantly Hispanic/Latino sample of youth whose families were affiliated with a youth-serving community organization that focuses on diversion programming (i.e., reducing teen pregnancy, increasing pursuit of higher education, and reducing substance use and gang involvement). We hypothesized that youth experiencing more anxiety and depression symptoms would report more barriers to mental health service use, and that these barriers would be both intrinsic and extrinsic.

## Method

### Participants

Participants were 277 adolescents (ages 11–18, *M*_*age*_ = 15.14, *SD* = 2.21) actively engaged in Project Youth OC, a community organization based in Santa Ana, California. Project Youth OC provides psychoeducation, diversion, and college/career preparation programs, with referrals received from schools, law enforcement, the probation department, the Student Attendance Review Board, and community events. Project Youth OC provides a uniquely robust and relevant sample for examining predictors of barriers to mental health service use due to the community’s documented socioeconomic challenges, gang-related violence exposure, and entrenched cultural barriers. Utilizing this distinctive sample enhances the external validity and practical applicability of findings, enabling the development of culturally informed interventions tailored to the specific needs of vulnerable Hispanic youth and their families. To be included in the study, adolescents were required to be current Project Youth OC participants capable of providing informed assent, along with written consent from a parent or legal guardian. The sample consisted of 52% females, and a majority (93.5%) identified as Hispanic or Latino.

### Procedures

All study procedures were developed according to best practices in conducting biobehavioral research in diverse community settings [13] and were approved by the Institutional Review Board at the University of California, Irvine. Project Youth OC staff members contacted parents via phone, email, or during in-person visits to the facility to invite them to participate in the study. Parents completed informed consent for their adolescent to participate in the study or indicated that their child was 18 years of age and could consent for themselves. If parents consented to have their child participate, they facilitated contact between the research team and the youth. In some cases, this involved inviting the youth to complete the study at an in-person data collection event at Project Youth OC; in others, this involved emailing or texting the link to the survey to participants to complete remotely. The first step of the survey provided the youth the opportunity to provide informed assent. Previous studies using this data have been published and provided additional details on study procedures as well as measures not included in the present analyses [14, 15].

### Measures

*Demographic characteristics.* Youth provided key demographic characteristics via self-report. Gender was assessed using the question, “What is your gender?” and could select from the following options: *Male*,* Female*,* Male to Female Transgender*,* Female to Male Transgender*,* Genderqueer/Gender non-conforming*, or *Other*. Parent English fluency was assessed using the question, “Do your parents speak English fluently?” and could select from the following options: *Yes both do*,* Just my mom*,* Just my dad*, or *No*,* neither of them do*. School level was assessed using the question “What grade are you in?” and participants could enter a whole number between 4 and 13 with 13 indicating that they were a “high school graduate or currently in college”.

*Early life adversity (ELA).* Youth self-reported their exposure to ELA via the Center for Youth Wellness Adverse Childhood Experiences Questionnaire [16]. Adolescents saw a list of nineteen adversities, including household dysfunction (e.g., you lived with a household member who served time in jail or prison), abuse (e.g., someone pushed, grabbed, slapped, or threw something at you), and neglect (e.g., more than once, you went without food, clothing, or a place to live, or had no one to protect you). Adolescents were asked to self-report the number of these adversities that they had ever experienced. Due to the sensitive nature of many of these adversities, item level data was not collected in order to protect the privacy of the youth and their families. Possible scores ranged from 0 to 19.

*Anxiety symptoms.* Youth self-reported their anxiety symptoms over the past month via the 41-item Screen for Child Anxiety Related Emotional Disorders (SCARED) [17]. Scores could range from 0 to 82 and scores greater than 25 indicated potential anxiety disorders. The internal reliability of the SCARED was excellent in the current sample, *α* = 0.95.

*Depressive symptoms.* Youth self-reported their depressive symptoms over the past month via the 30-item Reynolds Adolescent Depression Scale second edition (RADS-2) [18]. Total depression scores could range from 30 to 120 with scores greater than 77 differentiating respondents likely suffering from a depressive episode. The RADS-2 demonstrated excellent internal reliability in this sample, *α* = 0.95.

*Sleep disturbance.* Youth self-reported their sleep disturbances over the past month via the 18-item Pittsburgh Sleep Quality Index (PSQI) [19]. Potential scores on the PSQI range from 0 to 21, with scores of five or greater indicating the presence of insomnia or another sleep disorder. The internal reliability of the PSQI in our sample was similar to that observed in other adolescent samples where some sources of sleep disturbance are infrequent (e.g., use of medications) [20] but acceptable, *α* = 0.69.

*Barriers to mental health service use.* Youth self-reported their barriers to mental health service use via a modified version of the Barriers to Mental Health Services Scale Revised (BMHSS-R) [21]. The assessment begins by stating, “Listed below are potential reasons why people do not seek out mental health services,” and follows with a list of 34 items. Participants were asked to “indicate the extent to which you agree or disagree that the following barriers affect YOUR use of mental health services from 1 - Strongly Disagree to 4 - Strongly Agree.” The items reflect seven barriers that comprise corresponding subscale reflecting both intrinsic barriers (i.e., help-seeking, stigma, knowledge and fear of psychotherapy, finding a therapist, and belief that depression is normal) and extrinsic barriers (i.e., insurance/payment, physician referral, transportation). Internal consistency across all items was excellent, *α* = 0.93, though varied by subscale. Internal consistency was good for beliefs about inability to find a psychotherapist, *α* = 0.84, and transportation, *α* = 0.81. Internal consistency was acceptable for stigma, *α* = 0.78, knowledge and fear of psychotherapy, *α* = 0.73, belief that depressive symptoms are normal, *α* = 0.78, and cost, *α* = 0.74. Internal consistency was low for help-seeking, *α* = 0.59, and physician referral, *α* = 0.55.

### Data analysis

We first conducted a series of bivariate correlations or analyses of variance to determine whether any demographic, psychosocial, or symptom factors were associated with mean differences in self-reported barriers, as well as the extent to which barrier subscales correlated with one another. We then conducted a series of linear regressions predicting the total barriers and each barrier subscale as a function of the demographic, psychosocial, or symptom factors that were associated with mean differences in that domain. All *p*-values < 0.05 are reported for comparison with the broader literature, and a *p*-value < 0.006 is considered statistically significant after correcting for multiple comparisons across the 7 subscales of the BMHSS-R.

## Results

### **Participant characteristics and bivariate associations with total perceived barriers**

Bivariate comparisons of self-reported barriers by psychosocial predictors are reported in Table 1. ELA and clinically significant depressive, anxiety, and sleep disturbance were associated with more reported barriers overall, all *p* < 0.001, and this pattern emerged for each individual subscale as well, *p* < 0.001. Gender minority status was associated more beliefs that depression is normal, *p* =.038, and participants who were in college or no longer in high school reported more cost-related barriers than their peers who were still in school, *p* = 0.005.

**Table 1:** Unadjusted mean differences (*F-*values) in barriers to mental health services by psychosocial factor (*n* = 277)

				Intrinsic					Extrinsic		
		% (*n*)	Total	HS	S	KF	FT	BDN	IP	PR	T
Barriers to mental health services [M(SD)]		100 (277)	79.99 (16.68)	10.23 (2.34)	10.03 (2.96)	12.14 (3.16)	11.73 (3.32)	10.93 (2.69)	7.22 (2.04)	6.46 (1.70)	11.57 (3.24)
Gender			1.05	0.39	0.86	0.18	1.83	3.32*	0.59	2.10	0.84
	Male	45.5 (126)									
	Female	51.6 (143)									
	Gender minority	2.9 (8)									
School			0.84	0.04	0.52	0.32	0.89	0.93	5.46**	0.14	0.30
	< 9th grade	30.7 (85)									
	High school	50.5 (140)									
	Post-high school	18.4 (51)									
Parent English Proficiency			0.50	0.44	2.07	0.37	0.17	0.08	0.06	0.06	0.40
	Neither	49.1 (136)									
	One	24.5 (68)									
	Both	26.0 (72)									
Adverse childhood experiences			16.61***	8.04***	7.61***	8.96***	6.75***	12.26***	10.34***	9.70***	9.71***
	None	27.8 (77)									
	1–3	35.4 (98)									
	4 or more	36.1 (100)									
Depression			45.05***	20.40***	16.10***	17.26***	23.06***	56.34***	14.38***	47.27***	19.97***
	Normal range	70.8 (196)									
	Elevated symptoms	25.6 (71)									
Anxiety			52.76***	10.62***	14.63***	16.90***	42.11***	57.45***	29.47***	29.97***	31.84***
	Normal range	53.1 (147)									
	Elevated symptoms	40.8 (113)									
Sleep disturbance			44.12***	16.70***	9.66**	20.77***	28.12***	29.74***	17.27***	37.70***	31.65***
	Normal range	48.7 (135)									
	Elevated symptoms	45.8 (127)									

***p <.001, **p <.01, *p <.05

*HS* Help-seeking, *S* Stigma, *KF* Knowledge and Fear of Psychotherapy, *FT* Finding a Therapist, *BDN* Belief that Depression is Normal, *IP* Insurance/Payment, *PR* Physician Referral, *T* Transportation

### Independent predictors of total perceived barriers

Results of regression analyses predicting self-reported barriers as a function of psychosocial predictors are summarized in Table 2. The combination of ELA, and clinically significant symptoms of depression, anxiety, and sleep disturbance accounted for 24% of total barriers, *R*^*2*^ = 0.24, *p* < 0.001, and having clinically elevated symptoms of anxiety, *p* < 0.001, and sleep disturbance, *p* = 0.008, were both independently associated with more barriers overall.

**Table 2:** Adjusted estimated associations [*b(SE*)] between psychosocial factors and barriers

		Intrinsic					Extrinsic		
	Total	HS	S	KF	FT	BDN	IP	PR	T
Gender	–	–	–	–	–	0.01 (0.28)	–	–	–
School	–	–	–	–	–	–	0.45 (0.18)*	–	–
Parent English	–	–	–	–	–	–	–	–	–
ELA	2.08 (1.25)	0.17 (0.20)	0.36 (0.26)	0.47 (0.27)	0.08 (0.27)	0.23 (0.21)	0.40 (0.17)*	0.004 (0.14)	0.33 (0.26)
Depression	4.85 (2.48)	0.76 (0.39)	0.89 (0.51)	0.51 (0.53)	0.38 (0.53)	1.36 (0.41)***	−0.01 (0.34)	0.92 (0.27)***	0.21 (0.52)
Anxiety	7.38 (2.17)***	0.21 (0.34)	0.71 (0.44)	0.63 (0.47)	1.79 (0.47)***	1.37 (0.36)***	0.81 (0.30)**	0.39 (0.23)	1.24 (0.46)**
Sleep disturbance	5.71 (2.23)**	0.61 (0.34)	0.21 (0.44)	0.98 (0.46)*	1.05 (0.46)*	0.35 (0.35)	0.42 (0.29)	0.65 (0.23)**	1.32 (0.45)**

***p <.001, **p <.01, *p <.05

*HS* Help-seeking, *S* Stigma, *KF* Knowledge and Fear of Psychotherapy, *FT* Finding a Therapist, *BDN* Belief that Depression is Normal, *IP* Insurance/Payment, *PR* Physician Referral, *T* Transportation

### Independent predictors of intrinsic barriers

There were no psychosocial factors that independently accounted for barriers with respect to help-seeking or stigma. The combination of ELA, and clinically significant symptoms of depression, anxiety, and sleep disturbance accounted for 11% of barriers related to knowledge and fear of psychotherapy, *R*^*2*^ = 0.11, *p* < 0.001, and having clinically elevated symptoms of sleep disturbance was associated with more barriers related to knowledge and fear of psychotherapy, *p* = 0.03. The combination of ELA, and clinically significant symptoms of depression, anxiety, and sleep disturbance accounted for 17% of barriers related to belief about inability to find a psychotherapist, *R*^*2*^ = 0.17, *p* < 0.001, and having clinically elevated symptoms of anxiety, *p* < 0.001, and sleep disturbance, *p* = 0.02, were both independently associated with beliefs about inability to find a psychotherapist. The combination of gender, ELA, and clinically significant symptoms of depression, anxiety, and sleep disturbance accounted for 24% of barriers related to perceptions of normality, *R*^*2*^ = 0.24, *p* < 0.001, and having clinically elevated symptoms of depression and anxiety were both independently associated with more barriers related to belief that depressive symptoms are normal, both *p*s < 0.001.

### Independent predictors of extrinsic barriers

The combination of school type, ELA, and clinically significant symptoms of depression, anxiety, and sleep disturbance accounted for 16% of cost concerns, *R*^*2*^ = 0.16, *p* < 0.001, and having more exposure to ELA, *p* = 0.02, being more advanced in school, *p* = 0.01, and having clinically elevated symptoms of anxiety, *p* = 0.006, were each independently associated with more cost concerns. The combination of ELA, and clinically significant symptoms of depression, anxiety, and sleep disturbance accounted for 20% of barriers related to physician referral, *R*^*2*^ = 0.20, *p* < 0.001, and having clinically elevated symptoms of depression, *p* < 0.001, and sleep disturbance, *p* = 0.005, were both independently associated with more barriers related to physician referral. The combination of ELA, and clinically significant symptoms of depression, anxiety, and sleep disturbance accounted for 16% of transportation barriers, *R*^*2*^ = 0.16, *p* < 0.001, and having clinically elevated symptoms of anxiety, *p* = 0.007, and sleep disturbance, *p* = 0.003, were both independently associated with more transportation barriers. The combination of ELA, and clinically significant symptoms of depression, anxiety, and sleep disturbance accounted for 11% of barriers related to knowledge and fear of psychotherapy, *R*^*2*^ = 0.11, *p* < 0.001, and having clinically elevated symptoms of sleep disturbance was associated with more barriers related to knowledge and fear of psychotherapy, *p* = 0.03.

The combination of ELA, and clinically significant symptoms of depression, anxiety, and sleep disturbance accounted for 17% of barriers related to belief about inability to find a psychotherapist, *R*^*2*^ = 0.17, *p* < 0.001, and having clinically elevated symptoms of anxiety, *p* < 0.001, and sleep disturbance, *p* = 0.02, were both independently associated with more beliefs about inability to find a psychotherapist. The combination of gender, ELA, and clinically significant symptoms of depression, anxiety, and sleep disturbance accounted for 24% of barriers related to perceptions of normality, *R*^*2*^ = 0.24, *p* < 0.001, and having clinically elevated symptoms of depression and anxiety were both independently associated with more barriers related to belief that depressive symptoms are normal, both *p*s < 0.001. The combination of school type, ELA, and clinically significant symptoms of depression, anxiety, and sleep disturbance accounted for 16% of cost concerns, *R*^*2*^ = 0.16, *p* < 0.001, and having more exposure to ELA, *p* = 0.02, being more advanced in school, *p* = 0.01, and having clinically elevated symptoms of anxiety, *p* = 0.006, were each independently associated with more cost concerns. The combination of ELA, and clinically significant symptoms of depression, anxiety, and sleep disturbance accounted for 20% of barriers related to physician referral, *R*^*2*^ = 0.20, *p* < 0.001, and having clinically elevated symptoms of depression, *p* < 0.001, and sleep disturbance, *p* = 0.005, were both independently associated with more barriers related to physician referral. The combination of ELA, and clinically significant symptoms of depression, anxiety, and sleep disturbance accounted for 16% of transportation barriers, *R*^*2*^ = 0.16, *p* < 0.001, and having clinically elevated symptoms of anxiety, *p* = 0.007, and sleep disturbance, *p* = 0.003, were both independently associated with more transportation barriers. There were no psychosocial factors that independently accounted for barriers with respect to help-seeking or stigma.

### Posthoc analyses

The primary goal of this study was to better understand whether specific psychiatric symptom domains (anxiety, depression, sleep disturbance) were associated with distinct barriers to service use. Adolescents in this sample were all referred to this community organization for clinical need. Therefore, additional analyses were conducted to determine whether there were systematic differences in self-reported barriers to mental health service use between the youth in the sample who were currently participating in a Project Youth OC program (*n* = 75), who had never participated in a program (*n* = 135), or who were alumni of a Project Youth OC program (*n* = 62). Participants who were currently enrolled in a Project Youth OC program reported more barriers related to depression symptoms being normal, *p* = 0.008, and more barriers related to transportation, *p* = 0.016. There were no other mean differences between youth who were and were not currently enrolled in a Project Youth OC program in reported barriers to service use, all *p*s > 0.10. The results of the models predicting the beliefs that depression symptoms were normal and transportation barriers did not change when this factor was added to the regression models. Further current participation in a Project Youth OC program was not a significant independent predictor of either of these barrier subscales when accounting for other factors, *p*s > 0.39.

Given associations between symptoms and specific barrier subscales that suggest a role for primary care and physicians, posthoc follow-up analyses were conducted to explore the self-reported health service utilization in the sample. In the present sample, 72% (*n* = 199) had visited a primary care physician in the past year, 75% (*n* = 208) had visited a dentist, 18% (*n* = 51) had been treated in an urgent care or emergency room, but only 20% (*n* = 56) had contact with a mental health professional. Barriers (total or by subscale) did not reliably differ between participants who had or had not seen their primary care physician in the past year, all *p*s > 14.

## Discussion

**Fig. 1:**
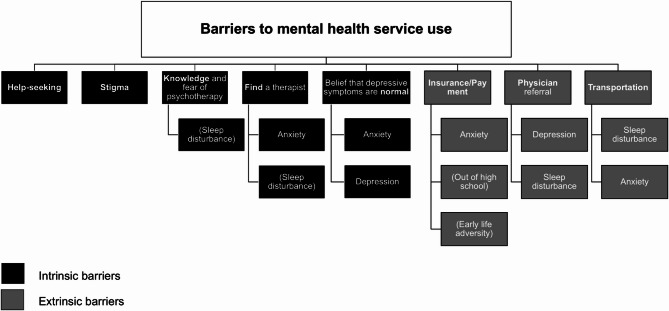
Summary of associations between psychosocial factors and barriers to mental health service use. Psychosocial factors shown were associated with each barrier domain in adjusted analyses at 95% reliability (*p* <.05). Psychosocial factors in parentheses denote associations that did not survive correction for the false discovery rate given comparisons across multiple outcomes

In this predominantly Hispanic/Latino sample of adolescents, having clinically significant symptoms of anxiety and sleep disturbances were most robustly and independently associated with total self-reported barriers to mental health service use. Anxiety was specifically associated with not knowing how to find a mental health service provider, believing that their symptoms were normal for their circumstances and community, and cost, whereas sleep disturbances were associated with not knowing what to expect from or fearing psychotherapy, not knowing how to find a mental health service provider, and not wanting to disclose their mental health concerns with a physician. While not associated with more barriers overall, having clinically significant symptoms of depression was associated with barriers related to believing that their symptoms were normal for their circumstances and community, and not wanting to disclose their mental health concerns with a physician. These findings underscore that psychiatric symptoms may shape adolescent’s perceptions of intrinsic (e.g., belief that depressive symptoms are normal, stigma, fear of psychotherapy) and extrinsic barriers (e.g., cost concerns, transportation, physician referral) and therefore access to care in distinct yet interrelated ways. A visual summary of these results is presented in Fig. 1.

These findings may reflect a bidirectional relationship in which symptoms and perceived barriers reinforce one another, perhaps particularly among adversity-exposed youth such as those represented in this study. This bidirectional pattern has been reported among adolescents with social anxiety disorder, such that symptoms of social anxiety exacerbate avoidance of help-seeking behavior [22]. Anxiety symptoms were, notably, the most robust predictor of barriers in this sample. Depressive symptoms and sleep disturbance were also reliably associated with several barrier subscales while sleep disturbance was associated with more barriers overall. Depressive symptoms and sleep disturbance may heighten withdrawal, mistrust, or emotion dysregulation, reducing adolescents’ ability to engage in help-seeking with providers [23]. While discomfort talking with physicians has been reported as a barrier in other samples [24, 25], very few have reported this pattern among adolescents and even fewer have linked this barrier to sleep disturbance.

Overall, there are at least three pre-existing approaches to reducing barriers that converge with the present findings: reducing reliance on physicians as gatekeepers to mental health services, improving adolescent trust with physicians, and integrated primary care. Adolescents can access mental health services in many ways, such as through specialty referrals from their primary care provider or through school-based providers (e.g., school psychologists, counselors, nurses) who are integrated within their learning community already. Qualitative research has identified several reasons talking with a physician may be a barrier among adolescents, which range from unfamiliarity with the provider, lack of trust, and lack of knowledge that their mental health symptoms are relevant to the care they receive from physicians [25, 26]. First, efforts to decrease reliance on physicians within the community to identify adolescents in need of mental health services while expanding efforts to train other adults with regular contact with adolescents such as teachers, caregivers, mentors, and coaches may be critical. There is already a growing evidence-base supporting the benefits of school-based mental health programs [27, 28]. Addressing adolescent discomfort discussing mental health concerns with physicians who regularly treat this population may also have important implications for integrated primary care. Evidence-based strategies for achieving this goal are well-established, such as the use of explicit confidentiality assurances [29, 30], though more are needed such as those that increase physicians’ confidence distinguishing between typical and clinical symptoms of behavioral disorders [6].

The present data also aligns with efforts to increase adoption of integrated primary care. Integrated primary care is a healthcare delivery model that combines primary care and behavioral health services within the same setting. Efforts to achieve integrated primary care have been justified in part by the need to increase access to mental health services among adolescents [31] and those efforts appear to be more effective than treatment as usual [32]. Notably, a majority of the present sample (72%) reported seeing their primary care physician in the past year but only a small proportion (20%) had interacted with a mental health professional during the same timeframe. Adolescents with clinically elevated symptoms in this high-risk, predominantly Hispanic/Latino sample reported more discomfort talking with physicians about mental health concerns, addressing which may maximize the potential benefit of integrated primary care. Additionally, the findings point to a possible sequencing or hierarchy of barriers, where intrinsic barriers may precede awareness of extrinsic ones, and therefore may be best targeted separately. For instance, if an adolescent does not recognize that their emotional distress may require support due to low mental health literacy, they may never get to the point of considering cost or transportation. In this way, intrinsic barriers such as normalization and lack of knowledge may act as gatekeepers, limiting the perceived need for care before structural constraints like cost or transportation even enter awareness. Indeed, two-stage interventions that target mental health literacy first have been recommended by meta-analytic evidence of clinical trials in youth [33]. It is also notable that adolescents’ discomfort talking to physicians was associated with more severe sleep disturbance. While sleep disturbance can be a symptom of depression, sleep disturbance can also occur for myriad other reasons and may be less stigmatized than something like depressed mood or anhedonia [34]. Despite the prevalence of sleep disturbance in pediatric populations, screening for sleep disturbance remains rare in pediatric primary care settings [35]. Yet, at least among young children, integrated primary care interventions targeting sleep have shown both feasibility and preliminary efficacy [36], making more widespread adoption of sleep screening of great clinical value.

Adolescents in this sample who were experiencing elevated symptoms of anxiety and sleep disturbance also report barriers to mental health treatment that center on not knowing how to find a mental health provider. This is a critical barrier to mental health service utilization that intersects insidiously with adolescents’ well-documented need for autonomy [37]. Community-level interventions that increase adolescents’ awareness of mental health service providers in their area, thus bypassing their need to actively pursue this information after symptoms arise, may facilitate their participation in preventative or early intervention services. Although minor consent laws vary, youth can still benefit from public health messaging that empowers them to understand options and initiate conversations about care.

Another notable pattern in this data was that more elevated symptoms of depression and anxiety were associated with perceptions that their symptoms were normal. Lower mental health literacy among adolescents has been well-documented as a barrier to mental health service use [25, 26]. Several studies have identified the lack of perception of need as a dominant barrier within clinical samples [38–40], particularly among those experiencing depression and anxiety symptoms. Adolescents may spend time with peers who experience the same symptoms and therefore perceive those symptoms as part of their adolescent development. If this is the case, expecting these youth to self-disclose to a healthcare provider about their symptoms is unrealistic. Efforts to integrate mental health providers into adolescents’ lives can be more effective, which has been shown repeatedly through school-based programs [27, 28]. Items in this subscale reflect participants’ degree of agreement with items such as “A lot of people feel sad and down,” and “It would be normal for me to be sad or down given the circumstances of my life.” While well-established among adolescents more broadly, this barrier may be more nuanced in this sample given the contexts in which they are living. Notably, participants in this sample reported exposure to far more adversity than is observed in the population; 36% of adolescents in this sample reported four or more adverse childhood experiences (ACE) compared to 12% in the general population [41]. While exposure to adversity was associated with this specific barrier in bivariate analyses (see Table 1), it was no longer associated with this barrier when accounting for depression and anxiety symptoms (see Table 2). Thus, increasing mental health literacy in ways that help adolescents identify their symptoms may help to reduce the psychiatric sequelae that follow adversity-exposed youth across the lifespan.

These results must be considered in the context of the study’s limitations. Limitations included the inability to determine whether different symptoms (e.g., anxiety vs. depression) lead to distinct patterns of service avoidance, which should be explored in longitudinal designs. These data were cross-sectional and therefore cannot speak to the causal relationship between psychiatric symptoms and barriers to mental health service engagement. Given the strong association between symptoms and perceived barriers overall, it is very likely that symptoms and barriers perpetuate one another over time, which a longitudinal study design could test. This dynamic may be amplified among subpopulations facing minority stress processes, such as the 2.9% who self-identified as gender diverse in this sample. Minority stress frameworks posit that distal stressors (e.g., discrimination) foster proximal processes (e.g., expectations of rejection) that can impede engagement with mental health providers [42, 43]. Assessing barriers to mental health service engagement through the lens of the minority stress model in a larger sample of gender diverse youth may help to identify effective mitigation strategies. Second, the participants in this study were predominantly Hispanic/Latino (93.5%) but likely varied significantly with respect to their cultural background or nationality. Santa Ana, CA, where the data was collected and where most participants lived, is composed mostly of individuals with heritage from El Salvador, Guatemala, Mexico, andthe U.S.A. Additional data on immigration background, nation of origin, as well as cultural traditions and beliefs would have been useful in better understanding perceived barriers to mental health service utilization in this sample. It is also important to acknowledge that the sequencing and salience of barriers may be influenced by culturally specific factors that were not measured in this study. For example, among Hispanic/Latino adolescents, barriers such as familial expectations around emotional self-reliance, concerns about legal status, language discordance between youth and caregivers, or cultural norms around privacy may shape how and when mental health needs are recognized and expressed. These factors may influence both intrinsic beliefs and perceived extrinsic risks, but were not directly captured in our measures. Future research should integrate culturally grounded frameworks to better understand how Hispanic/Latino youth navigate the process of seeking mental health support. Finally, psychiatric symptoms in this sample were assessed entirely via self-report questionnaire. While all three of the questionnaires used have well-established clinical thresholds with high clinical validity, it remains unknown whether participants with clinically-elevated symptoms were actually suffering from a psychopathology at the time of data collection. Adolescents are referred to Project Youth OC for a wide range of concerns from school truancy to substance use diversion; a more accurate characterization of their potential psychiatric conditions and comorbidities would aid efforts to reduce barriers to mental health service utilization among at-risk and symptomatic adolescents in a more targeted way.

Overall, the present study highlights that different psychiatric symptoms may be more closely associated with specific barriers to mental health treatment in a hard-to-reach, high-risk, and underrepresented sample of adolescents. Specifically, anxiety and sleep disturbance independently predicted more barriers as well as highlighted both intrinsic (finding a therapist, belief that depressive symptoms are normal) and extrinsic targets (insurance/payment, physician referral, transportation) to leverage within communities to mitigate unmet need.

## Data Availability

The data that support the findings of this study are available on request from the corresponding author. The data are not publicly available due to privacy or ethical restrictions.
